# Frequency-domain interferometry for the determination of time delay between two extreme-ultraviolet wave packets generated by a tandem undulator

**DOI:** 10.1038/s41598-023-37449-7

**Published:** 2023-06-25

**Authors:** Y. Hikosaka, T. Kaneyasu, S. Wada, H. Kohguchi, H. Ota, E. Nakamura, H. Iwayama, M. Fujimoto, M. Hosaka, M. Katoh

**Affiliations:** 1 Institute of Liberal Arts and Sciences, University of Toyama, Toyama, 930-0194 Japan; 2 SAGA Light Source, Tosu, 841-0005 Japan; 3 Institute for Molecular Science, Okazaki, 444-8585 Japan; 4 Graduate School of Advanced Science and Engineering, Hiroshima University, Higashi-Hiroshima, 739-8526 Japan; 5 Sokendai (The Graduate University for Advanced Studies), Okazaki, 444-8585 Japan; 6 Synchrotron Radiation Research Center, Nagoya University, Nagoya, 464-8603 Japan; 7 National Synchrotron Radiation Laboratory, University of Science and Technology of China, Hefei, 230029 China; 8 Hiroshima Synchrotron Radiation Center, Hiroshima University, Higashi-Hiroshima, 739-0046 Japan

**Keywords:** Ultrafast photonics, Optical spectroscopy

## Abstract

Synchrotron radiation, emitted by relativistic electrons traveling in a magnetic field, has poor temporal coherence. However, recent research has proved that time-domain interferometry experiments, which were thought to be enabled by only lasers of excellent temporal coherence, can be implemented with synchrotron radiation using a tandem undulator. The radiation generated by the tandem undulator comprises pairs of light wave packets, and the longitudinal coherence within a light wave packet pair is used to achieve time-domain interferometry. The time delay between two light wave packets, formed by a chicane for the electron trajectory, can be adjusted in the femtosecond range by a standard synchrotron technology. In this study, we show that frequency-domain spectra of the tandem undulator radiation exhibit fringe structures from which the time delay between a light wave packet pair can be determined with accuracy on the order of attoseconds. The feasibility and limitations of the frequency-domain interferometric determination of the time delay are examined.

## Introduction

Synchrotron radiation is a highly functional light used in various fields, including material science, biology, and medicine. Many experimental techniques have been developed based on the spatial coherence properties of radiation^1^. On the other hand, the use of the temporal coherence in time-domain interferometry has not been considered until recently. This is because, when dealing with a bunch of relativistic electrons distributing randomly in space, the generated synchrotron radiation lacks temporal coherence at wavelengths shorter than the bunch duration. However, the radiation associated with every single electron in the bunch holds perfect longitudinal coherence within the single light wave packet because the waveform of the light wave packet simply reflects the electron trajectory guided by the magnetic field without stochastic factor. Recently, the waveform of a single light wave packet has been observed by an autocorrelation measurement^2^ and a SPIDER method^3^, demonstrating longitudinal coherence within the single light wave packet. The availability of longitudinal coherence preserved in a single light wave packet contained in non-perfect temporal coherence light has been demonstrated by vacuum ultraviolet Fourier transform absorption spectroscopy using synchrotron radiation^4^ and by time domain interferometry using self-amplified spontaneous emission of a free electron laser^5^.

The longitudinal coherence of synchrotron radiation can be exploited in time-domain experiments based on light–matter interactions using a tandem undulator comprising two undulators placed in series^6,7^. A relativistic electron traveling in the tandem undulator sequentially generates two light wave packets, and the pair of the light wave packets naturally has mutual coherence. The longitudinal coherence between the two light wave packets was verified by an autocorrelation measurement^2^. Using a sequential interaction with a pair of light wave packets, two coherent quantum wave packets can be launched in matter. The interference between the quantum wave packets can be controlled by adjusting their phase difference. The phase adjustment can be achieved by tuning the time delay between the light wave packet pair. This scheme, called wave-packet interferometry^8^, is a standard method for coherent control experiments which have been developed in step with advances in laser technology.

The establishment of wave-packet interferometry using tandem undulator radiation was demonstrated by controlling the population^6^ and orbital alignment^7^ of the He Rydberg states. Moreover, it was shown that the femtosecond decay of an inner-shell excited state (e.g., 4d excitations in Xe atoms) can be tracked by sweeping the time delay^9^. While progress in laser technology has led to coherent control in the extreme ultraviolet (XUV) region^10–20^ and further in the soft X-ray region^21^, the use of tandem undulator radiation promises the extension of wave-packet interferometry toward even the hard X-ray region.

In wave-packet interferometry, controlling the time delay between light pulses provides coherent control. The temporal precision of the delay between light pulses must be better than or at least comparable to the optical periods of light; otherwise, the time-domain interference may be smeared out upon the overlap of different phases. Thus, higher precision in determining time delay is required in a shorter wavelength region. The required precision can be as short as attoseconds in the XUV region. In a tandem undulator setup, the time delay between the light wave packets can be adjusted using phase shifter magnets forming a chicane for the electron trajectory between the two undulators. An autocorrelation measurement of the radiation is a straightforward method for optically observing the time delay with attosecond precision, as its usefulness was proved in the UV region^2^. However, it is challenging to construct an optical interferometer for autocorrelation measurements in shorter wavelength ranges. Instead, the time delay between two light wave packets was determined in the XUV region using time-domain Ramsey interferometry based on light–matter interactions with similar time accuracy obtained in autocorrelation measurements^2^. However, the implementation of time-domain Ramsey interferometry is limited to XUV wavelengths where suitably narrow resonances exist.

In this study, we utilized frequency-domain interferometry to determine the time delay between an XUV light wave packet pair generated by a tandem undulator. The frequency-domain measurement of the time delay, which can be implemented with a conventional monochromator, has wide applicability to shorter wavelengths. The spectra recorded for the radiation from a tandem undulator exhibited fringe profiles, and the analysis of these spectral profiles provided time delays between light wave packet pairs. As the fringe features reflect the Fourier transform of the temporal waveform of the light wave packet pairs^22^, a reasonable time delay must be obtained from frequency-domain measurements. However, considering the attosecond precision required, it is not trivial to evaluate frequency-domain measurements under the finite emittance of electron bunches and a practical resolution of a monochromator. The present work clarifies the feasibility and limitations of frequency-domain measurements for the determination of the time delay of an XUV light wave packet pair.

## Results and discussion

**Figure 1:**
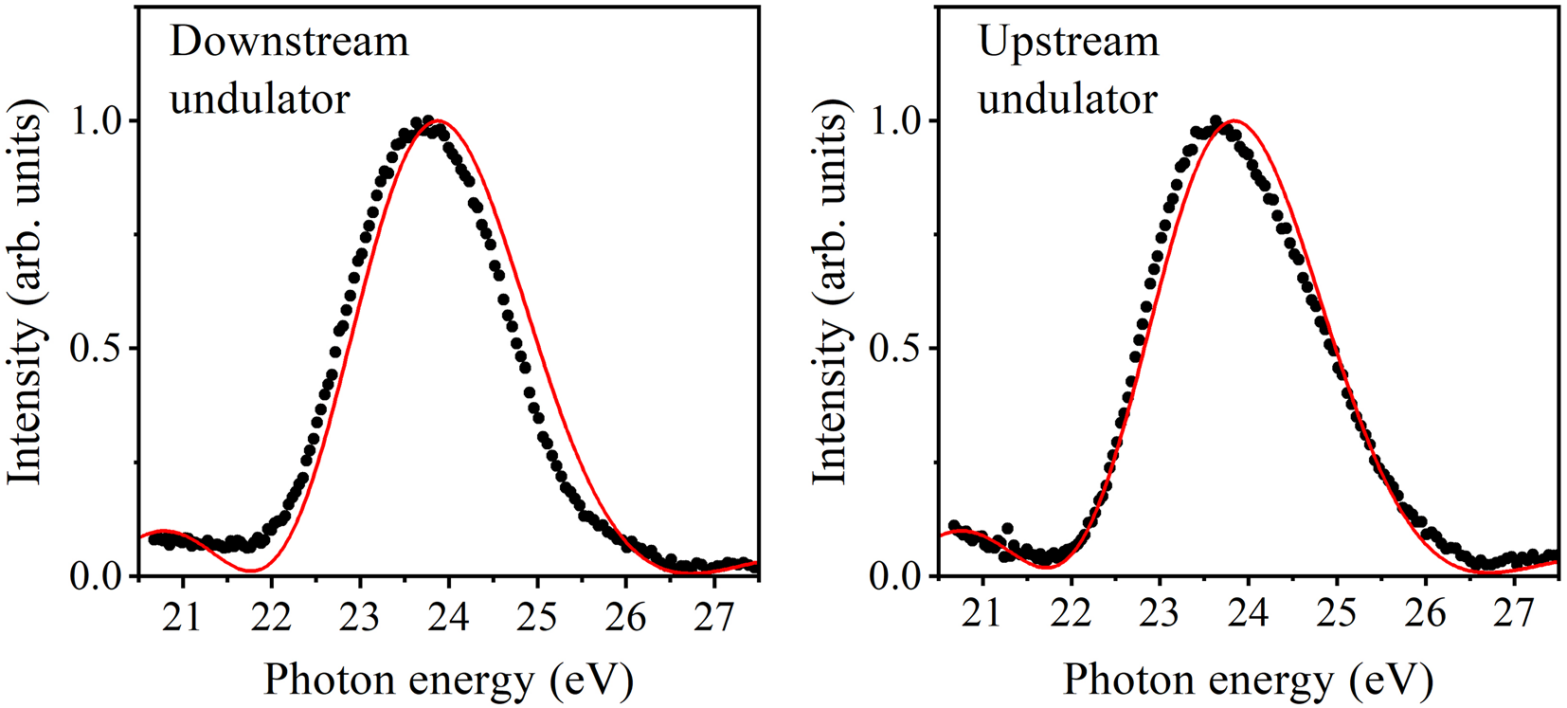
Radiation spectra of the individual undulators comprising the tandem undulator setup. The experimental and simulation spectra are shown by black dots and red lines, respectively, where the simulation spectra were convoluted with an experimental resolution of 0.25 eV (FWHM). The details of the tandem undulator are described in the Methods section**.**

Before focusing on the radiation from a tandem undulator, the radiation spectra of individual undulators in the tandem undulator configuration must be discussed. The central photon energy of radiation from one undulator was set to approximately 24 eV while opening the pole gap of the other undulator widely (200 mm) to make its radiation negligibly weak. The radiation spectra thus measured for the individual undulators are shown in Fig. 1. These spectra, as well as those presented later, were measured with a moderate resolution (FWHM of 0.25 eV) using a Seya–Namioka monochromator. The radiation spectra of the two undulators, which are similar to each other, show single bands whose widths were approximately 10% of the central photon energies. The spectra obtained by numerical simulations [see Methods] are compared to the experimental spectra in this figure. The simulation spectra reasonably reproduce the profiles and widths of the experimental spectra, although small adjustments of the simulation parameters can improve their correlation further.

**Figure 2:**
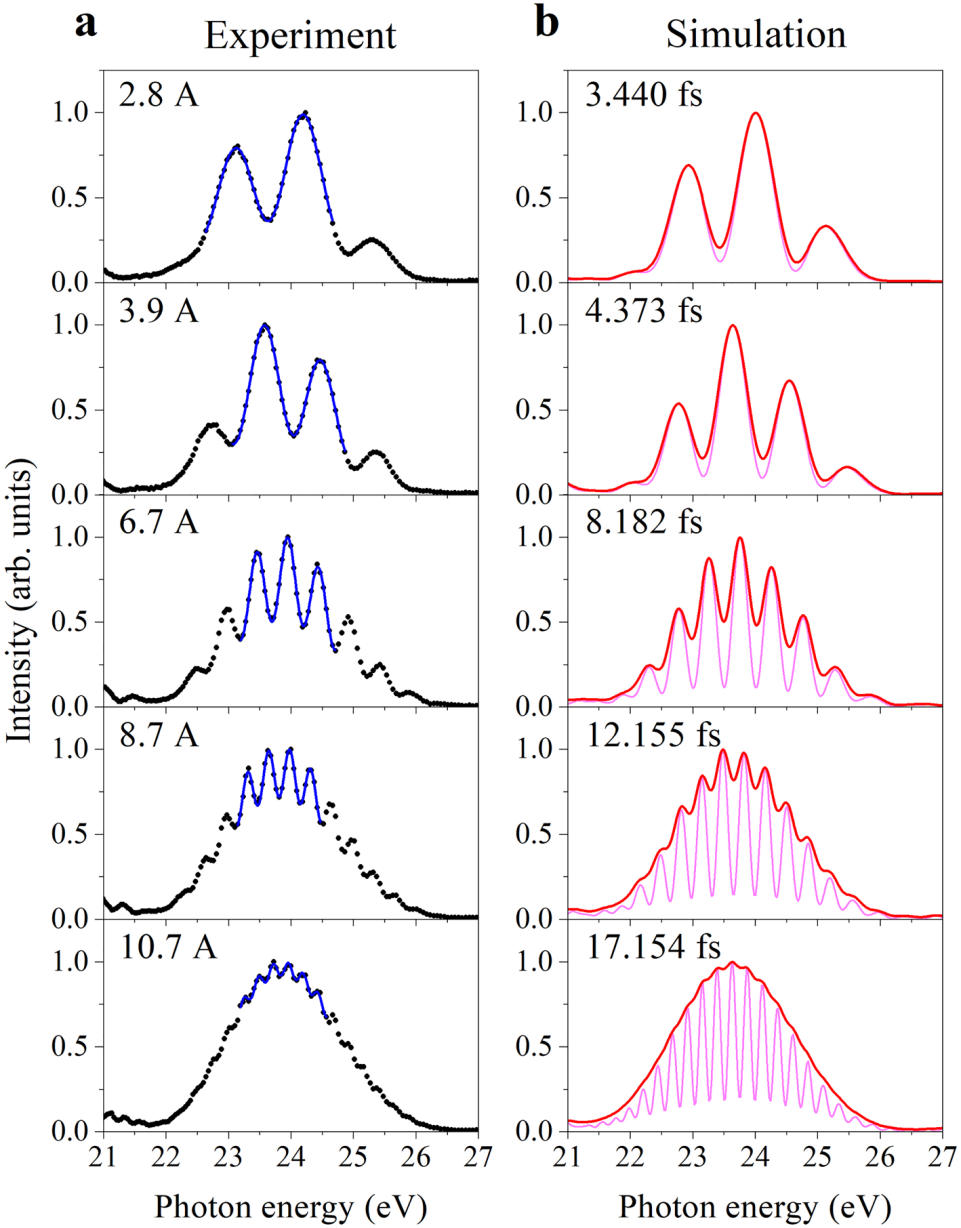
Frequency-domain spectra measured and simulated for the tandem undulator radiation at different phase shifter currents. (**a**) The experimental spectra are plotted with dots, and the fitting results with Eq. (1) are shown by blue line. (**b**) The simulation spectra with and without convolution about the monochromator resolution are shown in red and pink, respectively. Delays of the light wave packet pair derived from the temporal waveforms outputted by the simulation are indicated.

Next, both undulators were employed by setting the central photon energies of the radiations to approximately 24 eV. Figure 2a presents the frequency-domain spectra observed at different current conditions of the phase shifter magnets. Unlike the single undulator spectra in Fig. 1, these spectra exhibit fringe structures whose envelope delineated the radiation spectra of the individual undulators. With increasing the phase shifter current (i.e., with increasing delay time of the light wave packet pair), the number of fringes increases, whereas the fringe contrast decreases. These fringe structures indicate that the radiation of the tandem undulator has the form of a light wave packet pair in time^22^. Similar spectral profiles were observed by a previous measurement in the UV region^2^. The spectra obtained by the simulations for the conditions of the corresponding phase shifter currents are presented in Fig. 2b as pink curves. The valleys of the fringes in the spectra do not reach zero intensity, due to the finite emittance and energy spread of the electron beam^23^. The red spectra, obtained by convoluting the pink spectra with the monochromator resolution, reproduce the experimental features very well.

The fringe structures shown in Fig. 2 can be interpreted in terms of the optical interference between two light wave packets as follows. When a frequency component in one light wave packet and the same frequency component in the other light wave packet are in-phase, they constructively interfere with each other. The in-phase condition is fulfilled at the frequencies equal to the integer multiples of the reciprocal of the delay time $\tau$ between the light wave packet pair^23^. Thus, constructive interference appears at every $h/\tau$ in photon energy, where $h$ is Planck’s constant. As the time delay becomes longer, two neighboring photon energies that satisfy the in-phase condition become closer, resulting in finer fringes. Therefore, the time delay between the light wave packet pair can be determined from the $h/\tau$ spacings of the observed fringes. Precisely, because the envelopes of the fringe structures exhibit a Gaussian-like profile, the locations of the fringes in the observed spectra deviate from the $h/\tau$ spacings. To accurately determine the time delays from the fringe structures, we use a fitting function described as

1 $$I\left(E\right)\propto exp\left[-\frac{{\left(E-{E}_{0}\right)}^{2}}{2{\sigma }_{E}^{2}}\right]\times \left[1+F\mathrm{cos}\left(\frac{2\pi E\tau }{h}\right)\right]+{I}_{0},$$

where $E$, ${E}_{0}$, ${\sigma }_{E}$, $F$, ${I}_{0}$ are the photon energy, spectral center, spectral width, fringe contrast, and baseline, respectively. The term $F\mathrm{cos}\left(\frac{2\pi E\tau }{h}\right)$ represents the interference between the two undulator radiations whose profiles are assumed to be identical Gaussian functions^23–25^.

Prior to the determination of the time delays from the experimental spectra by fitting with Eq. (1), the reliability of the values obtained by the fitting procedure was inspected by its application to the simulation spectra convoluted with the monochromator resolution (red curves in Fig. 2b). The errors introduced by the fitting, i.e., the differences between the original delays in the time-domain simulation and the values obtained by the fitting to the convoluted spectra, were found to be within a few attoseconds when the spectral ranges to be fitted are appropriately chosen to those where fringe structures are clearly observed. It is verified here that, even for the fringe structures rather obscured due to the monochromator resolution, the fitting procedure itself does not introduce any pronounced error.

**Figure 3:**
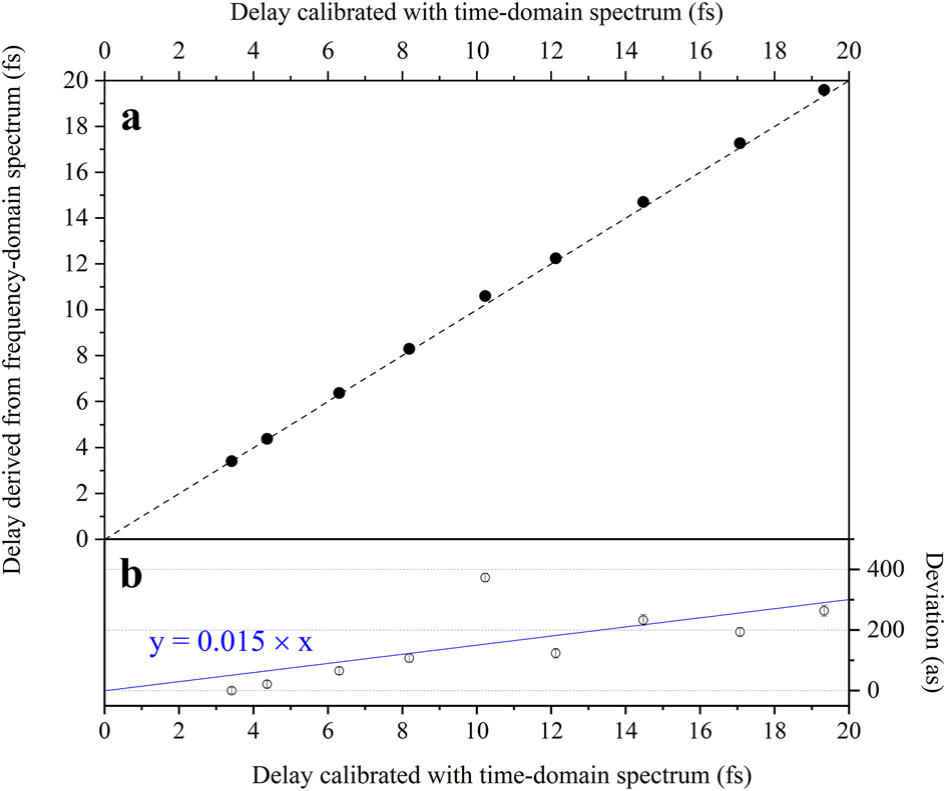
Delays between light wave packet pair, determined from frequency-domain spectra. (**a**) Values obtained by the frequency-domain determination are plotted against the time scale calibrated using the time-domain Ramsey spectrum. The broken line with a slope of 1 from the coordinate origin represents the values agreeing completely with the time-domain determination. (**b**) Deviations of the values obtained by the frequency-domain determination from the time scale calibrated by the time-domain determinations are plotted, where a linear curve fitting to the values is shown by a blue line.

The fitting with Eq. (1) was applied to the experimental spectra in Fig. 2a as well as those observed at other four different phase shifter currents. As shown in Fig. 2a, the fitting function well reproduces the fringe structures in the experimental spectra. Here the spectral ranges to be fitted were adjusted so that the residuals were small. As shown in Fig. 3a, the time delays determined by the fitting are plotted against the time scale calibrated using a time-domain Ramsey spectrum [see Methods]. Here, an onset to match with the delay time of the frequency-domain measurement at a phase shifter current of 2.8 A is added to the time scale from the time-domain determination (horizontal axis). The values obtained from the frequency-domain spectra lie fairly on a line with a slope of 1, implying that the fitting to the frequency-domain spectra properly determine the time delays.

The deviation between the values from the frequency-domain determination and the time-domain determination is shown in Fig. 3b. The deviation increases linearly as the longer time delay, rather than being randomly scattered. A linear curve fitting to the observed deviation gives a slope of 0.015; namely, the present frequency-domain determination of the delay scale gives an error of 1.5% assuming no error in the time-domain determination. A time precision less than the optical period of this undulator setting is thus achieved in the delay range of less than ~ 10 fs. Since practical measurements at short wavelengths, especially those targeting core–hole states with short lifetimes rarely require such a long delay range exceeding 10 fs, the delay time precision achieved by the frequency-domain determination is sufficient for practical applications. In fact, we have successfully tracked the fs decay of a 4d core–hole state of Xe using the current tandem undulator^9^.

The linear increase in the deviation shown in Fig. 3b suggests that the present frequency-domain determination was subject to a systematic error. The most probable error source is the energy calibration of the monochromator. A 1.5% reduction in the energy scale of the monochromator would result in the systematic error. Although the energy scale was calibrated to a higher degree of precision, it seems that the instability of the monochromator optics can introduce errors at this order of magnitude. Conversely, when a systematic error specific to the present measurement is eliminated, the precision by the frequency-domain determination can be improved over that demonstrated in this study.

## Conclusion

The time delay between an XUV light wave packet pair in the synchrotron radiation generated by a tandem undulator was determined by frequency-domain interferometry with accuracy on the order of attoseconds in the femtosecond range. The use of a conventional XUV monochromator of a moderate resolution under a practical synchrotron operation of finite emittance is effective enough for the frequency-domain interferometric determination. Frequency-domain measurements can be adopted to shorter wavelengths as far as hard X-rays. In addition to the high flexibility of applicable wavelengths, the method offers long delay time compared to time-domain interferometry based on light–matter interactions. While the maximum delay calibrated using time-domain Ramsey interferometry is limited by the lifetime of the resonant state whose population is monitored, resonant states in soft X-ray and hard X-ray ranges, associated with inner-shell excitations, have short lifetimes of femtoseconds or less. In contrast, frequency-domain interferometry is not subject to this limitation. On the future usages of light wave packet pairs in short wavelength regions, frequency-domain interferometry can be the primary method for delay time determination.

## Methods

### Experimental setup

**Figure 4:**
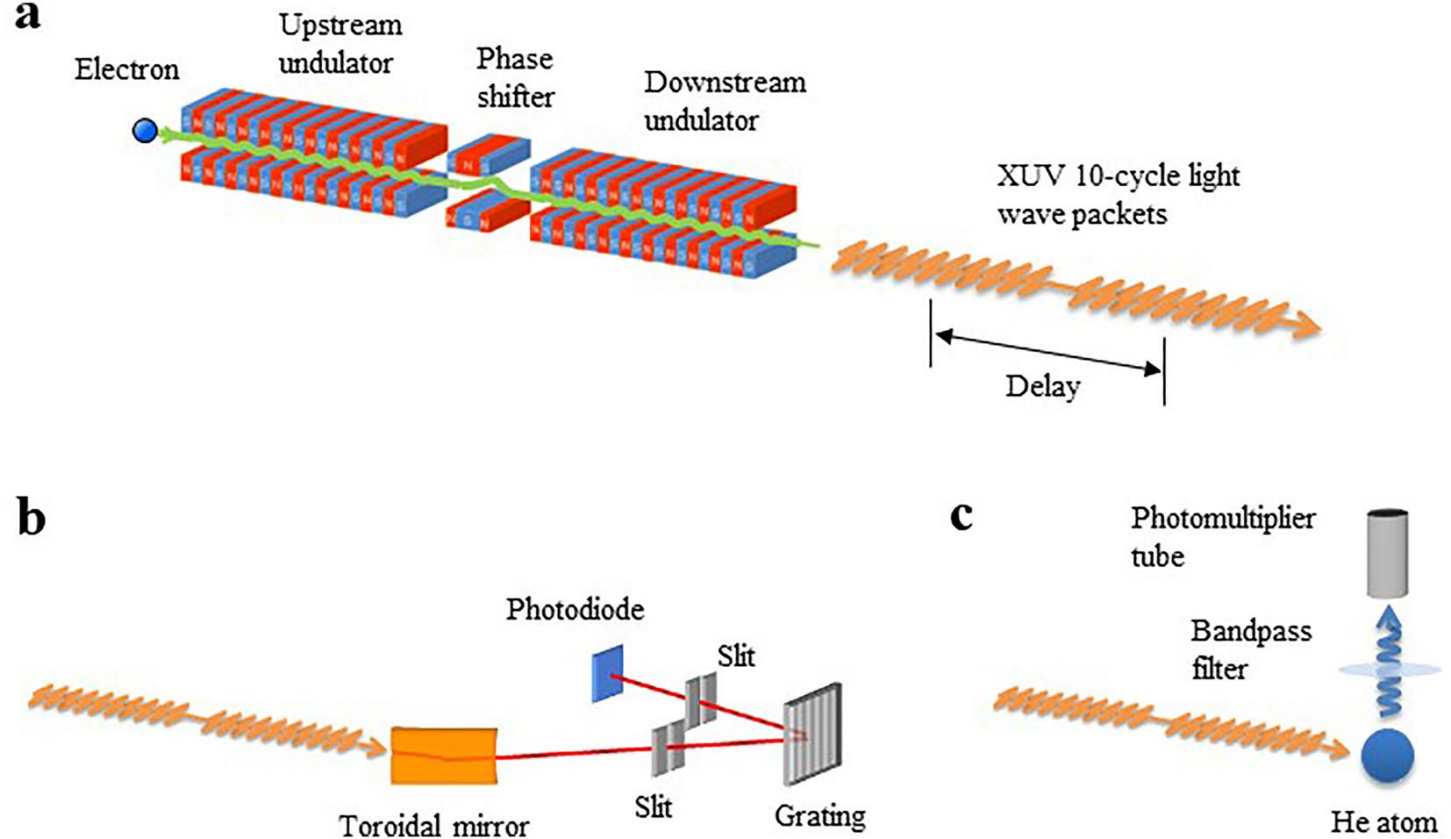
Schematic illustration of the tandem undulator and the experimental setups. (**a**) Tandem undulator setup. A relativistic electron passing through the undulators emits a pair of 10-cycle light-wave packets. The time delay between the wave packets is controlled at the attosecond level by the three-pole phase shifter placed between the undulators. (**b**) Experimental setup for frequency-domain measurements, using a 0.2-m Seya-Namioka monochromator with a 2400-lines/mm grating. (**c**) Experimental setup for time-domain interferometry. The 345-nm-wavelength fluorescence emitted from the 1s6p excited state in He atoms is detected by a photomultiplier tube equipped with a bandpass filter.

Experiments were conducted at the undulator beamline BL1U of the UVSOR-III synchrotron in Okazaki, Japan. The synchrotron was operated at energy of 750 MeV with an emittance of 17.5 nm·rad^26^, and the electron beam current was maintained to be around 10 mA during the experiments. In this condition, each electron bunch with a natural bunch length of 300 ps consists of ~ 10^9^ electrons. A tandem undulator, which comprised two identical APPLE-II undulators and a three-pole electromagnetic phase shifter placed in between (Fig. 4a), was employed. The period length and the number of periods for each undulator were 88 mm and 10, respectively. Both undulators were used in a horizontally linear polarization mode. The spatial center of the undulator radiation was cut by a 0.4-mm-diameter pinhole placed 9 m downstream from the midpoint of the two undulators.

The frequency-domain undulator spectra shown in Figs. 1 and 2 were measured by a 0.2-m Seya–Namioka monochromator with a 2400-lines/mm grating (Fig. 4b). The photon energy was calibrated by measuring the total fluorescence yield curve in the He 1s-to-Rydberg excitation range. The spectral resolution was estimated to be approximately 0.25 eV (FWHM). The intensity of the monochromatized light was observed by a Si photodiode (IRD AXUV100G) behind the exit slit.

### Numerical simulations

The simulation spectra presented in Figs. 1 and 2 were obtained by the synchrotron radiation calculation code SPECTRA^27^ using the near-field expression on the synchrotron radiation for the nominal machine parameters of the UVSOR-III synchrotron. The magnetic field distribution in the tandem-undulator section, used in the numerical simulation with SPECTRA, was separately calculated using the three-dimensional magnetostatics code RADIA^28^. The numerical simulation for a single relativistic electron passing through each undulator outputs its radiation as being a temporal waveform of 10-cycle oscillation. A frequency-domain spectrum for the undulator radiation was obtained by applying the Fourier transform to the temporal waveform. The finite emittance and the energy spread of the electron beam broaden the frequency-domain spectrum^27^, which was considered by a spectral convolution using the Gaussian distribution function reflecting the standard parameters of the UVSOR-III operation^26^.

### Time delay calibration by time-domain Ramsey interferometry

**Figure 5:**
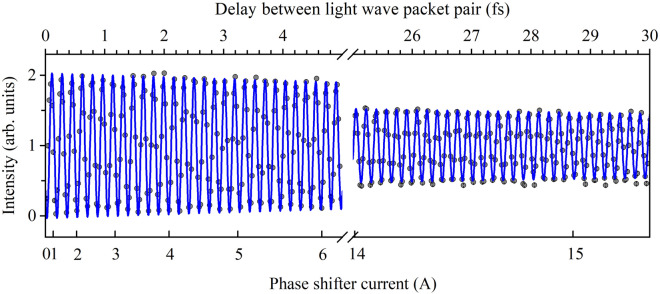
Time-domain Ramsey fringe spectrum. The fluorescence yield for the 1s → 6p excitation in He atoms, measured as a function of the phase shifter current, is plotted as dots. The time delay produced by the phase shifter was calibrated by fitting with a time-damped sinusoidal curve (blue curve) that oscillates at 170.8 as a period corresponding to the resonant frequency of the 1s → 6p excitation. The delay scale converted from the phase shifter current is shown on the top axis.

On the undulator settings to observe the frequency-domain spectra shown in Fig. 2a, the undulator radiations cover the resonance energies of 1s-to-Rydberg transitions in He. Therefore, the delay time between two light wave packets can be precisely determined using time-domain Ramsey interferometry by monitoring the populations of He excited states^2^, independently of the frequency-domain measurements. Figure 5 shows the Ramsey spectrum obtained by observing the fluorescence from the 1s6p excited state as a function of the phase shifter current. This spectrum was obtained by irradiating the undulator radiation directly to He gas without applying the monochromator optics (Fig. 4c). The 345-nm-wavelength fluorescence emitted in the decay of the 1s6p state into the 1s2s state was detected using a photomultiplier tube (Hamamatsu R6249P) equipped with a bandpass filter (Asahi Spectra HQBP350-UV). The Ramsey spectrum was normalized to the undulator radiation intensity monitored by a Si photodiode (IRD AXUV100G) placed behind the interaction zone.

The fluorescence intensity in Fig. 5 was measured at phase shifter currents that are expected to be equally spaced on the delay time scale. This is based on the knowledge that the delay time produced by the phase shifter magnet is approximately proportional to the square of the phase shifter current^23^. An oscillation at the definite period (170.8 as) corresponding exactly to the transition frequency of 1s → 6p is observed in the spectrum. The contrast of the oscillation gradually decreases with increasing time delay, due to the energy spread of the electron beam^23^. The oscillation of the definite period enables us to calibrate the delay time with higher precision than the oscillation period. The blue curve in Fig. 5 shows the result obtained using the time-damped sinusoidal equation, $I\left(t\right)\propto 1-\mathrm{exp}\left(-\gamma t\right)\mathrm{sin}\left({\omega }_{0}t+\varphi \right)$, fitted to the Ramsey spectrum^2^. Here, $t, {\omega }_{0},\gamma ,\mathrm{ and} \varphi$ are the delay time between the light wave packet pair, the transition frequency of 1s → 6p, the parameter reflecting the reduction in the fringe contrast, and the phase at the phase shifter current of 0 A, respectively, and the latter two are the fitting parameters. Based on the general agreement between the observed fringe structure and the fitting, the delay is calibrated over the wide delay time range of 0–30 fs. The precision of the calibrated delay was estimated to be ~ 20 as from the residuals between the experiment and the fitting curves. Note that, for the absolute delay time between the light wave packet pair, the onset delay resulting from the electron slippage in the undulators and the electron travel between the two undulators must be added to the determined delay.

## Data Availability

The data that support the findings of this study are available from the corresponding author upon reasonable request.
